# Psychopathological features of anorectic patients who dropped out of inpatient treatment as assessed by the Minnesota Multiphasic Personality Inventory

**DOI:** 10.1186/1751-0759-1-15

**Published:** 2007-07-25

**Authors:** Takehiro Nozaki, Satoko Motoyama, Tatsuyuki Arimura, Chihiro Morita, Chikako Koreeda-Arimura, Keisuke Kawai, Masato Takii, Chiharu Kubo

**Affiliations:** 1 Department of Psychosomatic Medicine, Graduate School of Medical Sciences, Kyushu University, 3-1-1 Maidashi, Higashi-ku, Fukuoka 812-8582, Japan

## Abstract

**Background:**

Anorexia nervosa often requires inpatient treatment that includes psychotherapeutic intervention in addition to physical and nutritional management for severe low body weight. However, such patients sometimes terminate inpatient treatment prematurely because of resistance to treatment, poor motivation for treatment, unstable emotions, and problematic behaviors. In this study, the psychopathological factors related to the personality of anorexic patients that might predict discontinuation of inpatient treatment were investigated using the Minnesota Multiphasic Personality Inventory (MMPI).

**Methods:**

Subjects were 75 consecutive anorectic inpatients who received cognitive behavioral therapy with a behavior protocol governing privileges in a university hospital based general (not psychiatric) ward. The MMPI was done on admission for all patients. A comparison was done of patients who completed the process of inpatient treatment, including attainment of target body weight (completers), and patients who dropped out of inpatient treatment (dropouts). Results: No significant differences between completers (n = 51) and dropouts (n = 24) were found in the type of eating disorder, age of onset, duration of illness, age, or BMI at admission. Logistic regression analysis found the MMPI scales schizophrenia (Sc), hypomania (HYP), deviant thinking and experience, and antisocial attitude to be factors predicting completion or dropout.

**Conclusion:**

Dropouts have difficulty adapting to inpatient treatment protocols such as our behavior protocol governing privileges because they have social and emotional alienation, a lack of ego mastery (Sc), emotional instability (HYP) and an antisocial attitude. As a result, they have decreased motivation for treatment, leave the hospital without permission, attempt suicide, or shoplift, which leads them to terminate inpatient treatment prematurely. Treatments based on cognitive behavioral therapy with a behavior protocol governing privileges should be carefully adopted for anorectic patients who exhibit the psychopathological elements identified in this study.

## Background

Anorexia nervosa (AN) with severe low body weight often requires psychotherapeutic treatment as well as physical and nutritional management on an inpatient basis. However, AN patients frequently terminate inpatient treatment prematurely because of resistance to treatment and lack of motivation to undergo treatment. In previous reports, patients who dropped out had psychological characteristics that included repression of anger [1], auto-aggressive behavior [2], impulsiveness [3], maturity of fears [4], low self-directedness and cooperativeness [1], and discrepant expectations of treatment between the patient and therapist [5]. However, these reports were unable to sufficiently clarify the reasons why anorectic patients so often drop out of treatment. Moreover, it is rather difficult to compare these dropout studies, because the definition of a dropout, treatment type (individual or family) and treatment setting (outpatient or inpatient) were different. To clarify the reasons for premature termination of treatment by anorectic inpatients who were treated with cognitive behavioral therapy, we investigated psychopathological factors, especially those related to personality, using the Minnesota Multiphasic Personality Inventory (MMPI). The MMPI has been shown to be useful for empirical research on the personality and general psychopathology of patients with eating disorders. We recently reported characteristics of the psychopathology of patients with prolonged AN as assessed by the MMPI [6]. In the same way, in this study we attempted to determine factors that would predict which anorectic patients might prematurely drop out of treatment, A comparison was done of patients who completed a course of inpatient treatment, including attainment of a target body weight about 90% of standard body weight, (completers) and patients who dropped out of inpatient treatment (dropouts).

## Methods

### Participants

The participants were 75 consecutive women admitted to Kyushu University Hospital from 2000 to 2003 and diagnosed with AN or an eating disorder not otherwise specified (EDNOS) according to the DSM-IV criteria [7].

We recommend inpatient treatment to outpatients whose body weight is extremely low or whose body weight or purging behavior does not improve in response to outpatient treatment. When a patient agrees to the treatment, a contract including a behavior protocol governing privileges is drawn up between the patient, their parents, and our medical staff. For emergency admissions, however, an agreement for inpatient treatment is obtained after the patient has gotten through the physical crisis. All participants gave informed written consent before entering the study.

### Characteristics of the therapeutic program

All patients received cognitive behavioral therapy with a behavior protocol governing privileges during hospitalization, a modification of the intensive, multimodal inpatient treatment for AN developed by Nozoe et al [8]. The characteristics of our inpatient treatment program are as follows: 1) inpatient treatment is done by a team consisting of physicians trained in psychosomatic medicine; which in Japan is a specialty in internal medicine, not psychiatry; certificated clinical psychologists and trained nursing staff; 2) patients have an approximately 60 minute personal interview twice a week in which poor cognition and behaviors are treated through counseling; 3) patients stay in a general ward in which other patients with internal medicine or psychosomatic disorders are treated, not a special eating disorder unit (such a treatment environment for eating disorder patients is popular in Japan); 4) family members are required to cooperate with the medical staff throughout the course of treatment ; 5) a target body weight is set at approximately 90% of standard body weight through consultation between the patient and the medical staff; 6) patients receive group therapy for social skill training to promote social adaptation.

### Therapeutic methods

Behavioral observation and physical examinations are done during the first two weeks of admission. In the initial phase of treatment, the patient is required to remain in bed, except when going to the toilet. Also, contact with family and friends, telephone calls, and the exchange of letters are prohibited at the beginning of treatment. As body weight increases, these limitations of behavior and communication are removed one by one based on our behavioral protocol governing privileges. If incremental improvement toward the expected body weight is not seen, privileges are deprived. This therapeutic method is similar to a traditional, strict program based on the principles of operant conditioning. When a patient has reached the target body weight, permission is given to make several overnight stays at their home.

The criteria for completion of our inpatient treatment are as follows: 1) consumption of meals of 2000 kcal/day or more and no observed binge eating or purging; 2) maintaining the target weight during a free food consumption period after achieving it, 3) normal eating and other social behavior during trial eating-out and home stays permitted after achieving target body weight; 4) successful establishment and maintenance of favorable personal relationships in the hospital ward. Whether or not these criteria are fulfilled is a team decision based on the clinical course coupled with the achievement of target weight.

### Testing instruments

All patients completed a battery of self-report questionnaires within one week after admission. The tests included an MMPI that has been standardized for use in Japan [9] and the Eating Disorder Inventory (EDI) and Eating Attitude Tests (EAT) to measure eating behavior and attitudes. We did not include results or discussion of the tests related to eating disorders in the present study because this study focuses on personality factors associated with dropping out. Our previous study showed that AN inpatients were often forced to discharge because of such behaviors as stealing or shoplifting and that their psychopathology was related to personality rather than that to the eating disorder. Moreover, other studies of dropouts have used EDI or EAT, whereas no studies of dropout from inpatient treatment based on MMPI were found.

The MMPI is a self-report inventory containing 566 descriptive statements for which patients indicate whether each statement is true or false [10]. The scores of the 566 items yield 1) three validity indices that suggest if the patient responded to the test items in a manner that would produce valid results, 2) 10 standard clinical and 13 Wiggins content scales [11] that suggest various features of psychopathology and personality functioning, 3) subscales of the clinical scales developed by Harris et al [12], which have come to be widely used, were routinely scored, and 4) 11 Lachar-Wrobel critical items, the content of which is judged to be indicative of serious psychopathology [13]. Raw scores on each scale were converted to T scores, and values 65 and greater are considered clinically significant. For general references concerning the MMPI, see Colligan and Offord [14] or Greene [15].

### Definition of dropping out

Dropping out was defined as any one-sided (patient or therapist) decision to discontinue treatment without completing the full schedule of treatment. Dropouts were classified by the phase at which they terminated treatment prematurely: 1) early phase: the period of behavioral observation before starting the behavioral protocol governing privileges (first two weeks of treatment); 2) middle phase: the period of treatment with the behavioral protocol governing privileges (after the third week but before reaching the target weight); or 3) late phase: the period after reaching the target weight. Moreover, we have described the direct cause of a patient quitting inpatient treatment by investigating the medical records of the dropouts.

### Statistical analysis

Comparisons of the demographic variables of the two groups were calculated using the student t-test or chi-square test. In the analysis of MMPI scores, univariate methods would tend to over-represent the number of between group differences because the scales within the MMPI are generally significantly correlated with one-another. In this study, we therefore chose multivariate analysis to determine between group differences in the MMPI clinical scales, content scales and critical items. Multivariate analysis of variance (MANOVA) was done to determine the possible differences in mean scale data among the groups. A MANOVA yielding a significant F was followed by a significant t by t-test for comparison of two groups or a significant F by ANOVA for three groups. Moreover, a Bonferroni correction for error in significance testing was applied, such that the α level for univariate t or F was determined by dividing 0.05 by the number of concurrent tests, because these t tests and ANOVA included multiple concurrent tests. For individual scales that differed significantly by each group with this Bonferroni corrected F, the protected least significant difference (LSD) procedure for comparison of three groups was performed. Logistic regression analysis was done to determine the factors of each scale most predictive of dropping-out. SSPS (12.0J for Windows) was used for statistical analysis. The results in the Tables are presented as means ± SD.

## Results

Table 1 shows the demographic and clinical characteristics of our patients with AN (n = 69) and EDNOS (n = 6) [see Additional file 1-T1]. No significant differences between completers (n = 51) and dropouts (n = 24) were found in the type of eating disorder, age of onset, duration of illness, age, or BMI at admission. When subjects with EDNOS were excluded, patients with restricting AN tended to be more likely to complete the inpatient treatment than binge eating/purging AN patients, but without significance (p = 0.08 by a chi-square test). Significant differences between completers and dropouts in body weight and BMI were seen at discharge (p < 0.0001). The duration of hospital stay was longer for completers than for dropouts (p < 0.0001).

A MANOVA revealed significant between group differences in the 13 validity and clinical scales of the MMPI (Wilks' lambda = 0.691, F(13,61) = 2.098, p = 0.027). Dropouts were significantly higher than completers on the Hs, Pd, Pa, Pt, Sc and Ma scales of the MMPI by t-tests with Bonferroni correction [p < 0.003 (0.05/13)] (Table 2) [see Additional file 1-T2]. MANOVA also revealed significant between group differences in the 13 Wiggins content scales (Wilks' lambda = 0.711, F(13,61) = 1.904, p = 0.047). Dropouts were significantly higher than completers on 10 of the 13 Wiggins contents scales by t-tests with Bonferroni correction [p < 0.004 (0.05/13)] (Table 3) [see Additional file 1-T3]. Significant between group differences were also found in the 11 Lachar-Wrobel critical items (Wilks' lambda = 0.457, F(11,62) = 2.573, p = 0.009). The dropouts had a higher score than completers for 9 of the 11 items by t-tests with Bonferroni correction [p < 0.004 (0.05/11)] (Table 4) [see Additional file 1-T4].

Logistic regression analysis with forward stepwise selection revealed that Sc, hypomania, deviant thinking and experience, and antisocial attitude were factors predicting dropout (Table 5) [see Additional file 1-T5]. Table 6 includes the subscales of Sc and shows that the dropouts had higher scores than completers for all subscales, social and emotional alienation, object loss, lack of ego mastery, and sensorimotor dissociation [see Additional file 1-T6].

Table 7 shows the reasons for dropping out. About 80% of all dropouts from inpatient treatment left during the middle phase treatment [see Additional file 1-T7]. There was no difference in the distribution of patients by phase between patients who left on their own initiative (group P) and patients whose treatment was terminated by their therapists (group T). Dropouts in the early phase (n = 4) had higher scores on many MMPI scales than patients who dropped out in the other phases (n = 20), but with no significant difference (data not shown). The reason for the lack of significance may have been that the sample size of patients in the early phase was too small. The direct causes of termination are as follows: reduced motivation for treatment (54%), including four patients (17%) who left the hospital ward or building without permission; shoplifting or theft of food (21%); suicide attempt or self-injury, mood instability, and violation of a rule of the hospital ward (each 8%). A significant difference in the direct cause of dropping out was found between group P and group T (χ^2 ^= 15.2, p < 0.01). A reduction of motivation was found more often in group P than in group T. Patients who left on their own initiative had a significantly higher family problems (FAM) score on the MMPI than those who were discharged by their therapist (64.8 ± 11.4 vs. 54.3 ± 11.8, p = 0.033, 95%CI= -20.13~-0.87) or completers (64.8 ± 11.4 vs. 48.6 ± 11.4, p < 0.001, 95%CI= -23.80~-8.65) by LSD procedure following MANOVA (Wilks' lambda = 0.559, F(26,120) = 1.601, p < 0.05) and ANOVA (F(2,72) = 9.378) with a Bonferroni correction [p < 0.006, (0.05/8)] (data not shown in Table).

## Discussion

MMPI was used to identify psychopathological factors that might predict which patients are at risk of dropping out of our cognitive behavioral therapy with a behavior protocol governing privileges for inpatient anorectic patients. The overall dropout rate of 32% found in our study was very similar to the rate of 31–33% documented by three recent studies [1,4,16]. As for demographic and clinical factors, some researchers have demonstrated that AN patients of the binge eating/purging type [3,16,17] of higher age at admission [18] and with a longer duration of illness [16,18] had a significantly higher rate of dropout from inpatient treatment. In contrast, we did not find differences in the subtype of AN, age at admission, or duration of illness between the dropouts and completers, consistent with the data of Zeeck et al. [4] for AN inpatients. The finding of no difference in the subtype of AN between completers and dropouts might be explained by our treatment strategy. When binge/purging behavior is found, we quickly counsel patients and implement a behavioral therapeutic intervention, by which they are encouraged to confront and overcome their problems. This may contribute to a reduction of patient resistance to change and reinforcement of the therapeutic relation for treatment, thus reducing premature treatment termination.

The psychopathology of dropouts included social and emotional alienation, lack of ego mastery, emotional instability, and an antisocial attitude. These characteristics make it difficult for patients to fit in well in an environment in which interpersonal relationships are important, such as during a hospital stay. Patients with an antisocial attitude often tend to break the rules of the hospital ward and easily act out. Also, patients predisposed to dropping out can be expected to have difficulty adjusting to a strict behavior protocol governing privileges because of their psychopathological characteristics. As a result, they have decreased motivation, leave the hospital without permission, attempt suicide, or shoplift, which leads them to drop out of inpatient treatment. Treatment based on cognitive behavioral therapy with a behavior protocol governing privileges was successful for many of our patients. However, it should be carefully adopted for anorectic patients who exhibit the psychopathological elements identified in this study because these patients have great difficulty accepting strict rules that limit their behavior. Moreover, treatment regimens must be developed to meet the specific needs of those patients. More family problems were found in dropouts who left because of therapist initiative than in either completers or dropouts who left because of patient initiative. Cooperation of the family is important to successful treatment, however family function is decreased under the conditions of severe family problems, which may result in a disincentive for treatment. Shoplifting and the theft of food accounted for almost half of our therapist initiated premature termination. We previously reported that the "hypomania" and "over-controlled hostility" subscales of the MMPI were risk factors for stealing behavior in AN patients [19]. "Over-controlled hostility" was not but "hypomania" was a psychopathological feature of our dropouts. "Hypomania" shows emotional instability and, especially when leaning toward the hypomanic state, a patient can be expected to have difficulty adjusting to a strict treatment protocol that includes limitations on behavior and communication and may easily start acting out. Careful consideration is required, therefore, in embracing this treatment program for patients with high "hypomania" scores. Patients terminated by their own initiative mainly because of decreased motivation for treatment, which included acting out in ways such as leaving the hospital without permission. To prevent dropout from inpatient treatment, we need to sufficiently explain the meaning of the behavior protocol governing privileges to both the patient and their family before admission and to confirm their motivation for treatment.

This study has some limitations. First, we did not evaluate all possible personality traits because we did not conduct a structured interview; therefore, the presence of personality disturbance was not assessed. Second, our treatment was done in a general ward of a university hospital, whereas most of the patients in European and American studies were treated in a unit specific for eating disorders. Because of the difference of environments, our results for premature termination may not be comparable to those of other studies. Third, although data showing reduced patient motivation was drawn from descriptions found in medical records, no quantitative measurement of motivation was done using a psychometrical test. Therefore, we can not conclusively report the extent to which a reduction of motivation influenced premature termination. Fourth, our results did not determine the prognosis of patents after discharge because we examined only dropout from inpatient treatment. It will be necessary to do more research on the long-term outcome of cognitive behavioral therapy with a behavior protocol governing privileges for AN patients.

## Conclusion

AN and EDNOS patients who discontinued inpatient cognitive behavioral therapy with a behavior protocol governing privileges had MMPI psychopathological features including schizophrenia (i.e., social and emotional alienation, and lack of ego mastery), hypomania, deviant thinking and experience, and an antisocial attitude. For patients who exhibit the psychopathological elements identified in this study, great care should be taken when adopting inpatient treatment with a behavior protocol governing privileges.

## Competing interests

The author(s) declare that they have no competing interests.

## Authors' contributions

TN conceptualized and designed the study, analyzed the data, interpreted the results, and drafted the manuscript. SM participated in the design of the study, collected and analyzed the data, and helped to draft the manuscript. All authors read and approved the final manuscript.

## Supplementary Material

Additional file 1 T1 tab: Table 1, Demographic and clinical features of the eating disorder patients; T2 tab: Table 2, MMPI-1 clinical scales; T3 tab: Table 3; MMPI-1 Wiggins content scales; T4 tab: Table 4, MMPI-1 Lashar-Wrobel critical items; T5 tab: Table 5, Logistic regression analysis for each MMPI scale; T6 tab: Table 6, Subscales of schizophrenia; T7 tab: Table 7, Subscales of schizophrenia. Click here for file
